# Enhanced absorption in all-dielectric metasurfaces due to magnetic dipole excitation

**DOI:** 10.1038/s41598-019-40226-0

**Published:** 2019-03-05

**Authors:** Pavel D. Terekhov, Kseniia V. Baryshnikova, Yakov Greenberg, Yuan Hsing Fu, Andrey B. Evlyukhin, Alexander S. Shalin, Alina Karabchevsky

**Affiliations:** 1 Photonics and Electrooptical Engineering Unit, Ben-Gurion University, Beer-Sheva, 8410501 Israel; 2 Ilse Katz Institute for Nanoscale Science & Technology, Ben-Gurion University, Beer-Sheva, 8410501 Israel; 3 Center for Quantum Information Science and Technology, Ben-Gurion University, Beer-Sheva, 8410501 Israel; 4 ITMO University, 49 Kronversky Ave, 197101 St. Petersburg, Russia; 5 Data Storage Institute, Agency for Science, Technology and Research (A*STAR), 2 Fusionopolis Way, #08-01, Innovis, Singapore 138634 Singapore; 6 Moscow Institute of Physics and Technology, 9 Institutsky Lane, Dolgoprudny, 141700 Russia; 7 Institute of Quantum Optics, Leibniz Universität Hannover, Hannover, 30167 Germany

## Abstract

All-dielectric nanophotonics lies at a forefront of nanoscience and technology as it allows to control light at the nanoscale using its electric and magnetic components. Bulk silicon does not experience any magnetic response, nevertheless, we demonstrate that the metasurface made of silicon parallelepipeds allows to excite the magnetic dipole moment leading to the broadening and enhancement of the absorption. Our investigations are underpinned by the numerical predictions and the experimental verifications. Also surprisingly we found that the resonant electric quadrupole moment leads to the enhancement of reflection. Our results can be applied for a development of absorption based devices from miniature dielectric absorbers, filters to solar cells and energy harvesting devices.

## Introduction

Absorbing and accumulating energy from light could enable smart sensors to work indefinitely. Therefore, many groups are joining their efforts in the academic and technological level to develop devices which will facilitate the energy absorption^1–3^. Even a few decades ago, silicon films have been used to construct solar cells and energy absorption devices^4,5^. Since then, there have been also discovered ways to use plasmonic^6,7^ and dielectric^8–11^ structures for the energy absorption and accumulation. Thus, the energy can be absorbed in a wide spectral range using low-loss dielectric materials if just would be possible to change their optical properties. The optical properties of dielectric metamaterials have been attracting significant attention in recent years mainly because of their possibility to support the excitation of both electric and magnetic multipole resonances^12–19^. Owing to this effect, all-dielectric metasurfaces are widely used for the controllable light manipulation, particularly to control phase^20,21^, polarization^22–24^ and transmission^25–27^. In addition, dielectric materials are in high demand due to their possibility to concentrate the electric field without Joule losses^28,29^. The opportunity to control the light scattering can be widely applied to develop optical nanoantennas^30–33^, radiation sources^34–37^, antireflective coatings^38,39^, cloaking techniques^40^, to improve MRI devices^41,42^, sensors^29^ and many others. The multipole decomposition approach^43,44^ is one of the widely-used methods to analyse the optical properties of dielectric metamaterials^12,45–49^. We, therefore, use the multipole decomposition to understand the contribution of multipoles to the absorption effect of the metasurfaces that we study.

**Figure 1:**
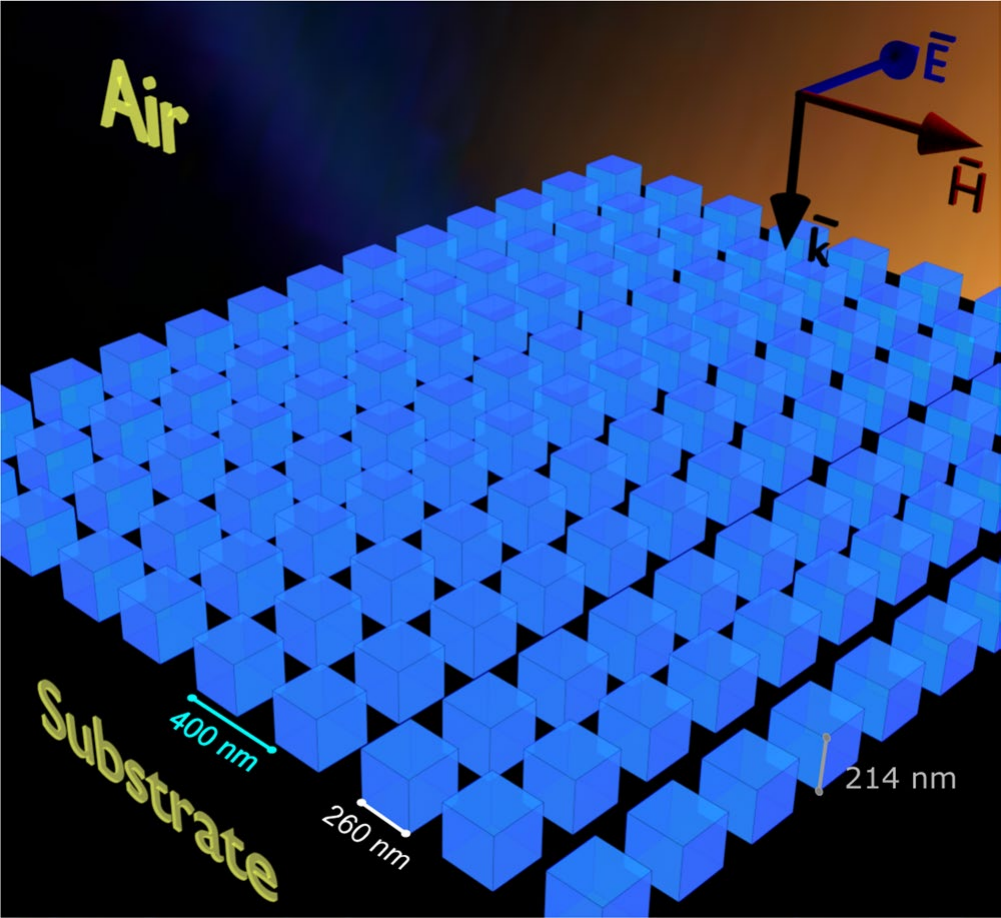
The proposed metasurface for efficient light absorption. It is constructed from silicon parallelepipeds with the height of 214 nm, the base edge of 260 nm and the lattice constant of 400 nm, on a substrate with refractive index of *n* = 1.51. The metasurface is embedded in air and illuminated by polychromatic light at normal incidence as indicated by $\bar{{\bf{k}}}$.

Here, we study the absorption of light in the silicon metasurfaces on the glass substrate depicted in Fig. 1. In our recent numerical studies we explored the properties of dielectric nanoparticles^50,51^. However, the behavior of particles in an array is not obvious even if their behavior as a single particle is well understood. Therefore, here we are taking a step forward and explore the optical properties of the collective response of the array of particles - the metasurfaces, to obtain the noticeable light absorption. In the current level of technology, the considered silicon parallelepipeds can be fabricated relatively easy using different techniques. We built the numerical model to analyse the transmission and reflection properties of such structures, as well as to calculate the multipole moments excited in every particle of the metasurface. During our analysis we found that the high-order multipole excitations directly affect the optical properties of the metasurface. Thus, for the described structure, the electric quadrupole (EQ) moment can be associated with enhanced reflection and the magnetic dipole (MD) moment provides the extraordinary absorption. Moreover, the interference between the total electric dipole (TED) and MD moments leads to the realization of the Kerker-type effects in the considered metasurface. In addition to the numerical calculations, here we describe the experimental results at the nanoscale. We fabricated the periodic metasurface starting from an amorphous silicon thin film using the focused-ion beam (FIB) technique and analysed its properties. For this, we first deposited the amorphous silicon layer of the height of 214 nm and then created the periodic metasurface of the parallelepipeds with the square base of *D* = 260 nm and the lattice constant of 400 nm. The transmission, reflection, and absorption spectra of the fabricated metasurface were experimentally measured and explained by the multipole decomposition approach. In addition, the experimental results show the good agreement with theoretical predictions.

## Theoretical Background

To analyse the multipole behavior in the periodic metasurface, we use the same theoretical background as for the single particles we considered in ref.^44^ however numerically we construct the model with the boundary conditions dictating the periodic nature of the metasurface. Here, we integrate the light induced polarization over a single element of the infinite periodic metasurface to calculate the multipole contributions to the scattering electric field amplitude ${{\bf{E}}}_{0}^{sca}$. In our multipole decomposition approach, we use the electric dipole (ED) moment **p**, the magnetic dipole (MD) moment **m**, the electric quadrupole (EQ) moment $\hat{Q}$, the magnetic quadrupole (MQ) moment $\hat{M}$, the toroidal dipole moment **T** and the electric octupole moment $\hat{O}$^44^. The electric and toroidal dipole moments which are the multipole moments of the first and third orders, respectively, can be treated as the total electric dipole (TED) moment ${\bf{D}}={\bf{p}}+\frac{i{k}_{d}}{{v}_{d}}{\bf{T}}\equiv {\bf{p}}+\frac{i{k}_{0}}{c}{\varepsilon }_{d}{\bf{T}}$, where ${v}_{d}=c/\sqrt{{\varepsilon }_{d}}$ is the speed of light in the medium with relative dielectric permittivity *ε*_*d*_.

To acquire the multipole decomposition spectrum, we consider the total multipole contributions to the scattering amplitude ${{\bf{E}}}_{0}^{sca}({\bf{n}})$ in all directions **n** (**n** is a unit vector directed along **r**, which is the radius-vector to the observation point)^52^:

1 $$\begin{array}{c}{{\bf{E}}}_{0}^{sca}({\bf{n}})\simeq \frac{{k}_{0}^{2}}{4\pi {\varepsilon }_{0}}([{\bf{n}}\times [{\bf{D}}\times {\bf{n}}]]+\frac{1}{{v}_{d}}[{\bf{m}}\times {\bf{n}}]+\frac{i{k}_{d}}{6}[{\bf{n}}\times [{\bf{n}}\times \hat{Q}{\bf{n}}]]\\ \,\,\,\,+\frac{i{k}_{d}}{2{v}_{d}}[{\bf{n}}\times (\hat{M}{\bf{n}})]+\frac{{k}_{d}^{2}}{6}[{\bf{n}}\times [{\bf{n}}\times \hat{O}({\bf{n}}{\bf{n}})]]),\end{array}$$

where *k*_0_ and *k*_*d*_ are the wave numbers in vacuum and in surrounding medium respectively, *ε*_0_ is the vacuum dielectric constant.

Considering the multipole decomposition spectrum with this approach, we can associate the multipole response of every particle in the array with the total optical response of the entire metasurface. Note that the Eq. 1 describes the scattering amplitude in homogeneous medium with *ε*_*d*_. In the next sections, we present the numerical and experimental results describing the broadband absorption effect obtained with the designed metasurface. For qualitative estimations of the multipole contributions to the transmission and reflection spectra we use Eq. 1 with **n** = (0,0,1).

## Results

### Numerical results

**Figure 2:**
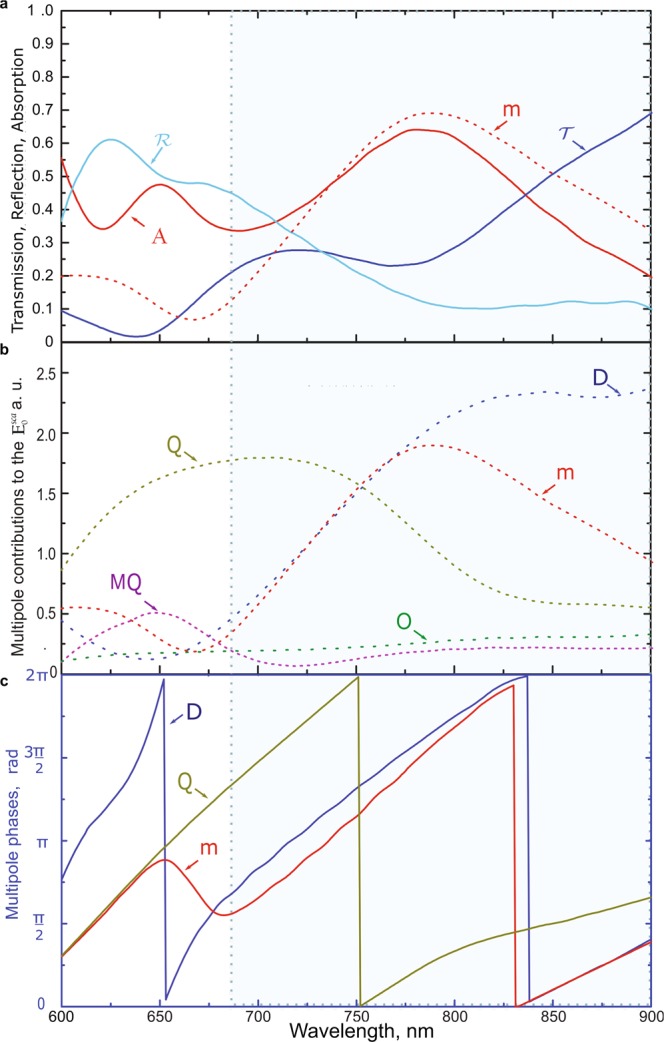
(**a**) The calculated transmission ${\mathscr{T}}$, reflection ${\mathscr{R}}$ and absorption ${\mathscr{A}}$ coefficients for the silicon metasurface on the borosilicate glass with the structure parameters as indicated in Fig. 1. The contribution of the MD moment is presented (similar to the **b**) as the dashed line for the convenient comparison. (**b**) The absolute values of the multipole contributions to the electric field amplitude $({{\bf{E}}}_{0}^{sca})$ scattered by the metasurface. (**c**) The phase spectra of the absolute values of the contributions of the TED, MD and EQ moments calculated for **n** = (0, 0, 1). The area of the broadband absorption by the metasurface (compared to the thin film) is enclosed in the blue box bounded by the dashed line.

To explore the wide-band absorption effect, we consider the metasurface which consists of the parallelepipeds with the square base edge of 260 nm, the height of 214 nm and the lattice constant of 400 nm. First, we calculate the transmission, reflection, and absorption coefficients (Fig. 2a). Second, we perform the multipole analysis (Fig. 2b). Here, the metasurface is illuminated from the superstrate. However, according to the numerical calculations, the change of illumination direction has a negligible effect on the transmission, reflection, absorption spectra and multipole contributions to the scattered filed.

Figure 2 shows the calculated transmission, reflection, and absorption spectra. The absorption peak at the wavelength of *λ* = 800 nm corresponds to the dip in the transmission spectrum. At *λ* = 725 nm, there is the well-pronounced absorption gap which corresponds to the transmission peak around this wavelength. To analyse the contribution of the multipole moments in the absorption broadening effect, we calculated the multipole decomposition of the electric field amplitude ${{\bf{E}}}_{0}^{sca}$ presented in Fig. 2b. Figure 2c shows the calculated multipole phases of the total electric dipole **D**, magnetic dipole **m** and electric quadrupole **Q** moments in the considered structure. The area of the broadband metasurface absorption is enclosed in the dashed bluish box.

Let us consider the region around *λ* = 725 nm. One can note that the TED and MD moments have similar contributions to the electric field amplitude ${{\bf{E}}}_{0}^{sca}$, which leads to the transmission peak due to the well-known Kerker effect^51,53^. The multipole phases are presented in Fig. 2c. The phases of the TED and MD moments tend to merge starting from *λ* = 675 nm. The similar phase of these moments is the second condition to achieve the Kerker effect. However, one can see the broad electric quadrupole resonance area around the same wavelength (*λ* = 675 nm). Due to the EQ excitation, the transmission in this range still does not increase crucially for this structure. The reflection coefficient in Fig. 2 in the range of 600 nm ≤ *λ* ≤ 775 nm can be associated with the dominating EQ resonant contribution to the scattering process. The MQ resonance in this area is not dominant enough to entail the strong interference and to provide the additional suppression of the backscattering in the considered structure^54^. In the region around *λ* = 775 nm, the transmission spectrum experiences a dip. This dip appears together with the well-pronounced absorption peak in this area, so the reflection does not crucially increase. It could be noted that the mentioned absorption peak is associated with the excitation of the MD moment resonance at the same wavelengths region as in Fig. 2. The absorption in the region of the lower wavelengths appears due to the usual properties of silicon in the visible range. In the region of 825 nm ≤ *λ* ≤ 900 nm, the absorption decreases, and despite the magnitudes of the TED and MD moments become different, their phases become similar. Together with the decrease in the EQ moment contribution, the Kerker-type effect is realized and therefore the transmission increases.

#### Influence of the substrate

**Figure 3:**
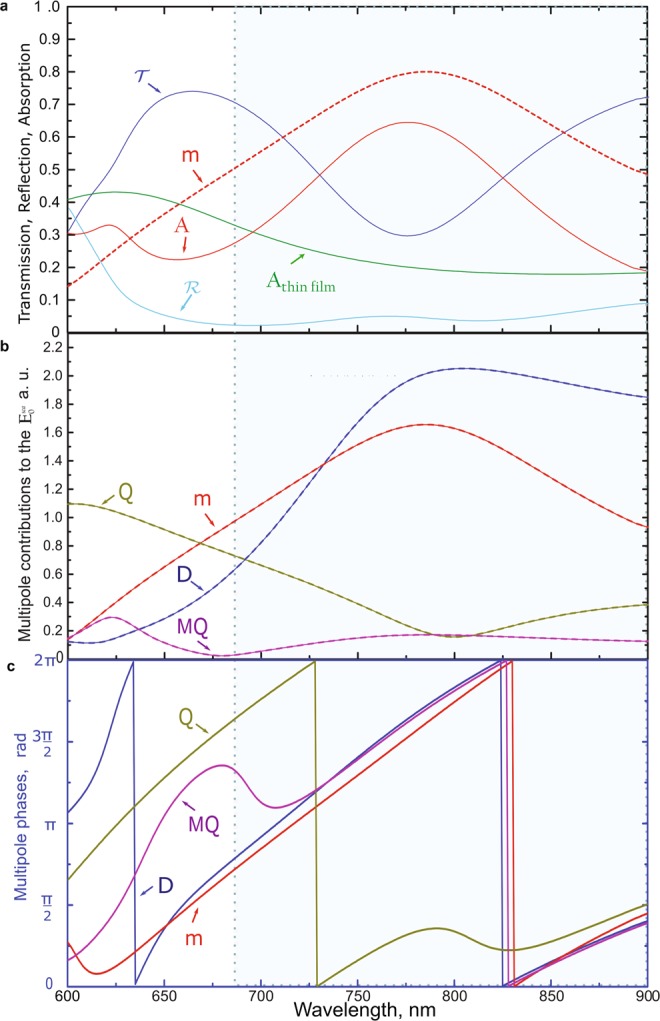
(**a**) The calculated transmission ${\mathscr{T}}$, reflection ${\mathcal R}$ and absorption ${\mathscr{A}}$ coefficients for the silicon metasurface in air with the structure parameters as indicated in Fig. 1 (but the substrate here has *n* = 1). *A*_*thin film*_ is presented to compare the metasurface absorption and the absorption of the thin film with the similar dispersion. The contribution of the MD moment is presented (similar to the Fig. 3b) as the dashed line for the convenient comparison. (**b**) The absolute values of the multipole contributions to the electric field amplitude ${{\bf{E}}}_{0}^{sca}$ scattered by the metasurface in air. (**c**) The phase spectra of the absolute values of the contributions of the TED, MD, EQ and MQ moments calculated for **n** = (0, 0, 1). The area of the broadband absorption of the metasurface (compared to the thin film) is enclosed in the dashed blueish box.

To study the influence of the glass substrate, we analysed the structure made of the same nanoparticles but in this case, they are embedded in air. In Fig. 3 one can note that the EQ moment resonant region experiences a blue shift and hence the reflection at 600 nm ≤ *λ* ≤ 750 nm dramatically decreases compared to the Fig. 2. Due to the lower contribution of the EQ moment to the light scattering process, the pronounced transmission peak appears at *λ* = 675 nm. This effect can be obtained due to the in-phase interaction of the TED and MD moments as one can see in Fig. 3c. However, the wide absorption peak due to the MD moment resonance remains almost unchanged at *λ* = 775 nm. It is interesting to note that the small MQ resonance at *λ* ≈ 630 nm also experiences the blue shift.

**Figure 4:**
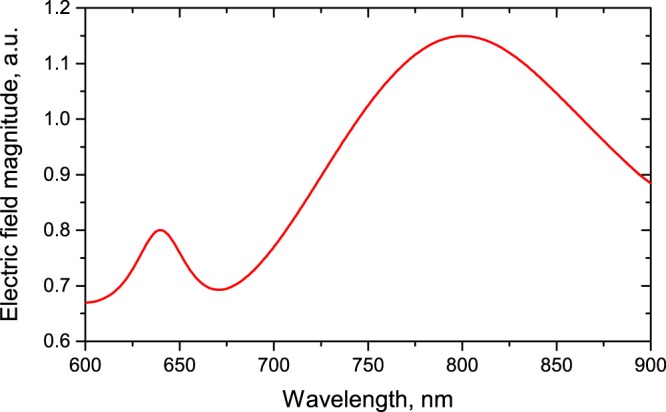
The calculated spectrum of average value of full electric field inside the nanoparticle for the silicon metasurface in air with the structure parameters as indicated in Fig. 1 (but the substrate here has *n* = 1).

We note that the presence of the substrate does not crucially influence the absorption peak at *λ* = 775 nm. In Fig. 3a, we also compare the absorption of the considered metasurface and the absorption of the thin silicon film with the same dispersion spectrum. It can be seen that the absorption of continuous silicon film does not experience dramatic changes in the considered wavelength range; however, it increases more than twice at *λ* = 775 nm. Importantly, the broadening of the absorption effect is realized entirely due to the metasurface parameters and it is not associated with the natural properties of silicon. Worth noting that spectral region of MD resonant excitation corresponds to the enhancement of electric field concentration inside each parallelepipedal particle constructing the metasurface (Fig. 4). Figure 4 confirms that the electric field is more efficiently concentrated inside the nanoparticle for MD resonant excitation. Thus, such an increase in broadband absorption and electric field concentration inside the particle is an artificial property of the designed silicon metasurface.

### Experimental results

**Figure 5:**
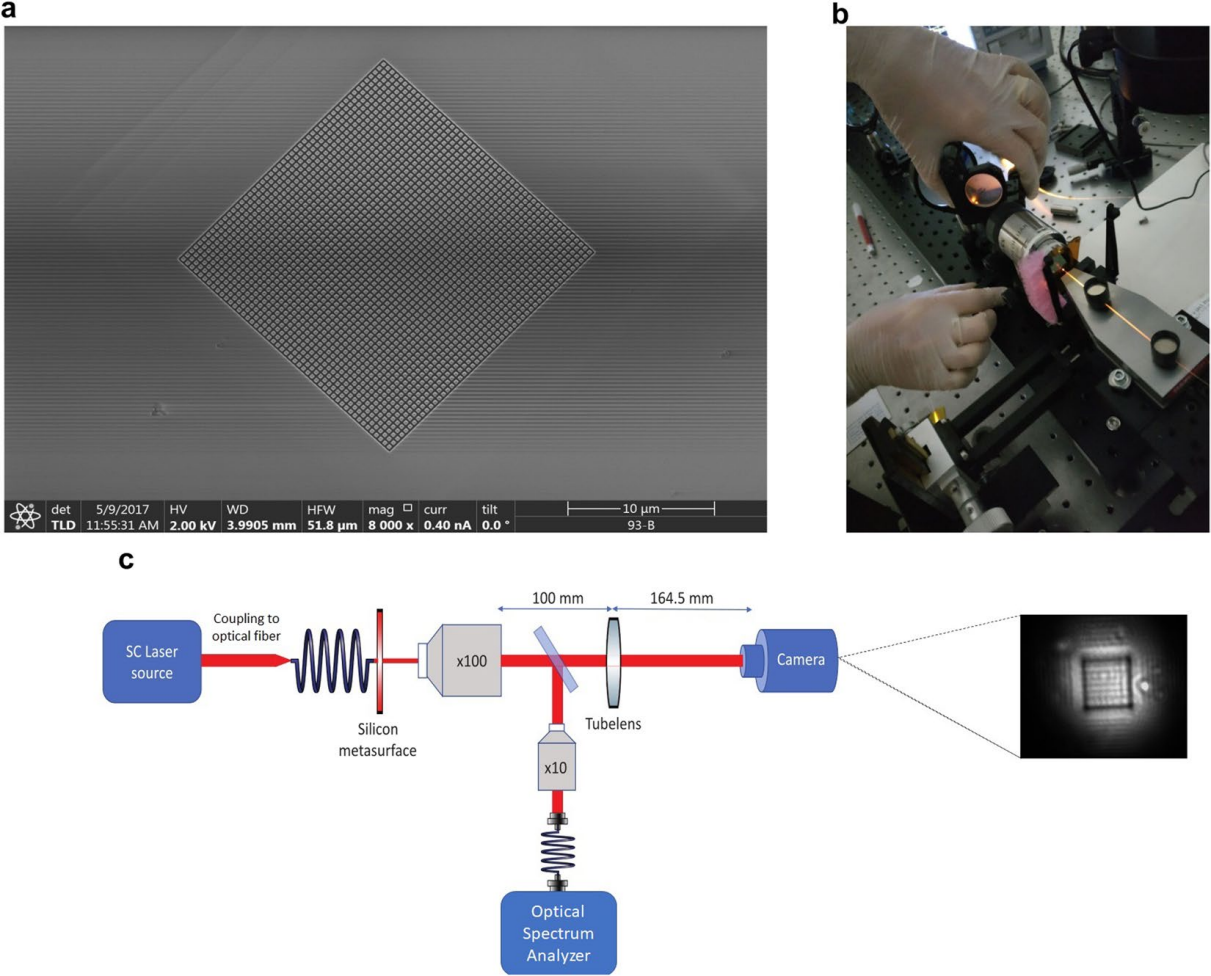
(**a**) The scanning electron micrograph (SEM) of the parallelepipedal dielectric metasurface fabricated by a focused ion beam milling, top view. (**b**) The photograph of the home-made experimental setup for the transmission measurements constructed in BGU. (**c**) The schematics of the experimental apparatus. The broadband source is coupled to the single mode optical fiber. The fiber illuminates the metasurface with the divergence angle of 7.4°. The transmitted light is split with the 50:50 beamsplitter for (1) the imaging of the metasurface and (2) the collection of transmitted light. The collection is performed with the optical spectrum analyser connected to the multimode optical fiber (MMF). The transmitted light was coupled into the MMF with the ×10 objective.

**Figure 6:**
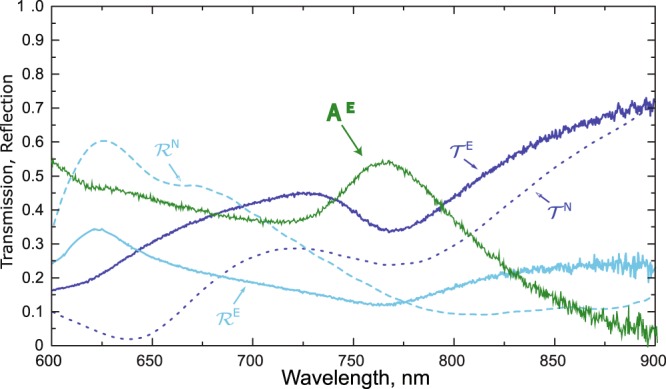
The comparison of the experimentally measured (the smooth curves labeled by the superscript E) and the numerically calculated (the dashed curves labeled by the superscript N) transmission ${\mathscr{T}}$ and reflection ${\mathcal R}$ spectra of the metasurface. ${{\mathscr{A}}}^{{E}}$ represents the experimentally measured absorption, calculated as 1 − ${{\mathscr{T}}}^{{\mathscr{E}}}$ − ${ {\mathcal R} }^{{\mathscr{E}}}$.

To prove the concept of the engineered absorption to be enhanced and broad by the silicon metasurface, we milled the thin film silicon on the area of 20 × 20 *µm* and the thickness of 214 nm using the focused ion beam technique. The scanning electron micrograph (SEM) of the fabricated metasurface shows the fabricated pattern (Fig. 5a). To perform the experimental measurements, we constructed the home-made setup at BGU shown in Fig. 5b. Schematics of the experimental setup is shown in Fig. 5c. The transmission spectrum of the sample was measured in the wavelength range of 600–900 nm^55^ and shown in Fig. 6. For reflection, the same objective lens (5x) was used for light incidence and collection.

We note that the transmission peaks and dips qualitatively coincident with the numerical predictions. The higher transmission through the fabricated sample can be explained by weaker excitation of electric quadrupole moment EQ due to the fabrication tolerances and the imperfect parallelepipedal shape of the milled meta-atoms. This reason also explains the lower reflection in the area of resonant excitation of EQ moment (the comparison of the measured and calculated reflection spectra is presented in Fig. 6b). The reflection measurements have been performed using the customized micro-spectrometer setup for the transmission and reflection measurements. The combination of the experimentally measured transmission and reflection spectra also proves the realization of the absorption peak around *λ* = 775 nm.

## Conclusion

To conclude, here we proposed the nano-scale metasurface for energy harvesting applications. We demonstrated that despite having no magnetic response as a bulk, the patterned silicon metasurface experiences magnetic response leading to the enhancement and broadening of the absorption. We found that the metasurface made of the parallelepipeds supports excitations of the multipole moments up to the third order. Comparing to nanodisk resonators, particles of rectangular shape ensure an additional degree of freedom in terms of the geometry to tune the optical properties of the whole structure. We noticed that the multipole behavior in the metasurface unit cells is related to the transmission properties of the whole metasurface. We showed that the excited electric quadrupole contributes to the abrupt decrease in overall transmission. However, the resonant magnetic dipole dictates the appearance of the absorption peak. It appears that the silicon metasurface absorbs up to 65% of light in the region where silicon is the low-loss material. In fact, as we showed the thin film experiences absorption twice lower in the same region. We also showed that the interference between the total electric dipole and the magnetic dipole moments leads to the enhanced transmission effect. Our results pave the road toward new generation of energy harvesting devices at the nanoscale just due to the light-manipulation with the high-order multipole excitations.

## Methods

### Numerical modelling

The numerical calculations have been performed with the RF module of the COMSOL Multiphysics commercial package using the finite element method (FEM). The substrate influence has been taken into account using the two-step numerical model. The multipole excitations have been analysed using the multipole decomposition approach considering the irreducable Cartesian representations of the multipole moments.

### Fabrication

**Figure 7:**
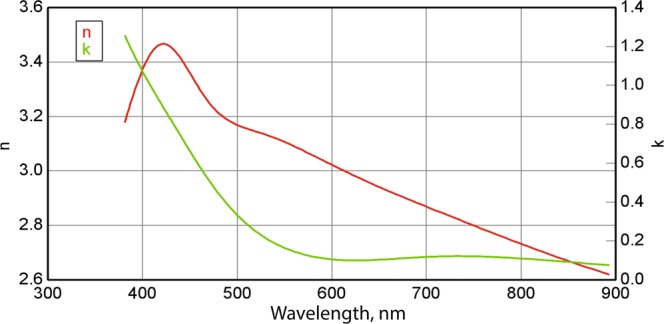
Spectra of n and k of the deposited amorphous silicon film.

The silicon film has been deposited in BGU using the system equipped with the 3 kW 4 pocket e-Gun, the thickness monitoring, the sample heater and the indirect temperature monitoring. The dispersion of n & k parameters measured with this system is presented in Fig. 7. The metasurface has been milled at Technion-Israel Institute of Technology, using the the dual beam focus ion beam (FIB) machine “Helios nano-lab G3” manufactured by Thermo Fisher Scientific (FEI). The sample was milled with gallium cations. Since it is known that the glancing incidence milling produces fewer larger effect on the silicon lattice strains as compared to the normal incidence milling, we milled our sample at normal incidence and at low energy to minimize the lattice damage. In addition, according to the numerical calculations, the slope angle due to the FIB milling does not crucially affect the properties of the structure, because the electric field mostly concentrates in the particle volume.

### Experiment

The ellipsometry measurements have been conducted in BGU with the QDI alpha-SE spectrosopic ellipsometer. The experiment in BGU has been conducted with the home-made setup for the transmission measurements. The sample was illuminated from the close distance *d*_*exp*_ ≈ 0.05 mm through the single mode fiber using the supercontinuum white light source “Fianium WhiteLase”. Transmitted light has been collected with the x10 optical objective and coupled to the multimode fiber which is in turn was connected to the optical spectrum analyser “Yokogawa AQ6370D”. The schematics of this home-made setup is shown in Fig. 5c. The experimental measurements of the transmission and reflection in DSI have been conducted with the customized micro-spectrometer setup.
